# Magnetic ground state of an individual Fe^2+^ ion in strained semiconductor nanostructure

**DOI:** 10.1038/ncomms10484

**Published:** 2016-01-28

**Authors:** T. Smoleński, T. Kazimierczuk, J. Kobak, M. Goryca, A. Golnik, P. Kossacki, W. Pacuski

**Affiliations:** 1 Institute of Experimental Physics, Faculty of Physics, University of Warsaw, Pasteura 5, 02-093 Warsaw, Poland

## Abstract

Single impurities with nonzero spin and multiple ground states offer a degree of freedom that can be utilized to store the quantum information. However, Fe^2+^ dopant is known for having a single nondegenerate ground state in the bulk host semiconductors and thus is of little use for spintronic applications. Here we show that the well-established picture of Fe^2+^ spin configuration can be modified by subjecting the Fe^2+^ ion to high strain, for example, produced by lattice mismatched epitaxial nanostructures. Our analysis reveals that high strain induces qualitative change in the ion energy spectrum and results in nearly doubly degenerate ground state with spin projection *S*_*z*_=±2. We provide an experimental proof of this concept using a new system: a strained epitaxial quantum dot containing individual Fe^2+^ ion. Magnetic character of the Fe^2+^ ground state in a CdSe/ZnSe dot is revealed in photoluminescence experiments by exploiting a coupling between a confined exciton and the single-iron impurity. We also demonstrate that the Fe^2+^ spin can be oriented by spin-polarized excitons, which opens a possibility of using it as an optically controllable two-level system free of nuclear spin fluctuations.

## Abstract (web summary)

Iron Fe^2+^ ions embedded in semiconductor matrix usually have zero magnetic moment in the ground state. Here, the authors show theoretically and experimentally that a nearly doubly degenerate magnetic ground state is produced when iron ions are subjected to strain in epitaxial quantum dots, suggesting they could be used in spintronics and solotronics.

## Graphical abstract

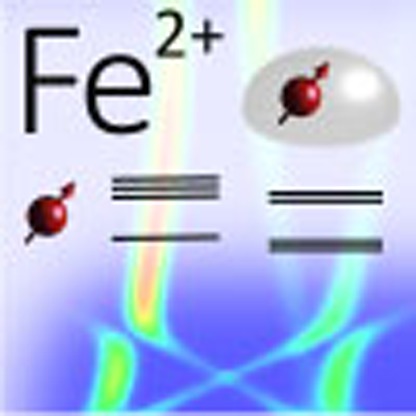

Spin configurations of transition metal ions in various host semiconductors have been well-established already a few decades ago^1^,^2^,^3^,^4^,^5^,^6^,^7^,^8^. It has been found that ions such as Cr^2+^(*d*^4^), Mn^2+^(*d*^5^), Co^2+^(*d*^7^) exhibit nonzero spin in their ground states, which makes them useful in spintronics^9^,^10^. For example, spin degree of freedom can be utilized in memories based on giant magnetoresistance^11^,^12^ or to process information using spin-transistor^13^. A fundamental requirement for manipulation of the spin is the availability of at least two different spin levels in the considered system. However, the ground state of the Fe^2+^(*d*^6^) ion in zinc-blende or wurtzite II–VI semiconductors like ZnS, ZnSe, CdTe or CdSe has been found to be nondegenerate^14^,^15^,^16^,^17^,^18^,^19^,^20^,^21^,^22^ with average spin 〈*S*_*z*_〉=0 and thus termed nonmagnetic^23^. To induce Fe^2+^ magnetic moment, high-magnetic field has to be applied, as for Van Vleck paramagnets^24^,^25^.

The physics of the transition metal ions has been recently brought back into the spotlight owing to the possibility to access the properties of single dopants^26^,^27^,^28^,^29^,^30^,^31^,^32^,^33^. The main motivation of optoelectronics with single-dopant atoms—solotronics^34^—is related to its potential for quantum information processing. Among other achievements, optical orientation^35^,^36^,^37^,^38^,^39^,^40^, readout^26^,^27^,^32^,^41^ and coherent precession^42^,^43^ of a single magnetic ion spin were demonstrated. Current development of the field benefits greatly from the fundaments of the early findings. However, the physics of the transition metal ions in semiconductor nanostructures goes far beyond the limits established in the earlier works on bulk materials.

Here we demonstrate that, contrary to the well-established knowledge on a Fe^2+^ ion in the semiconductor matrix, it is possible to qualitatively change its low-field behaviour from nonmagnetic to magnetic (by magnetic we consider a state which splits linearly upon application of a magnetic field of magnitude equivalent to ion-carrier exchange interaction, that is, of up to 1 T). In particular, we show that the magnetic behaviour of the Fe^2+^ ion can be induced by placing such an ion in a highly strained nanostructure. In order to elucidate this fact, we analyse the Fe^2+^ energy spectra for the cases of weak and strong strain, showing a clear hierarchy of the energy scales, relevant both to zinc-blende and wurtzite structures. The magnetic behaviour of the Fe^2+^ ion is experimentally evidenced by analysing the magnetic field dependence of the photoluminescence spectrum of an individual CdSe/ZnSe quantum dot (QD) containing a single Fe^2+^ impurity. The nonzero spin ground state of the Fe^2+^ ion even at low magnetic field opens the possibility of using it as a two-level system in quantum information technology^9^.

## Results

### Fe^2+^ energy spectrum in bulk and in a strained nanostructure

**Figure 1:**
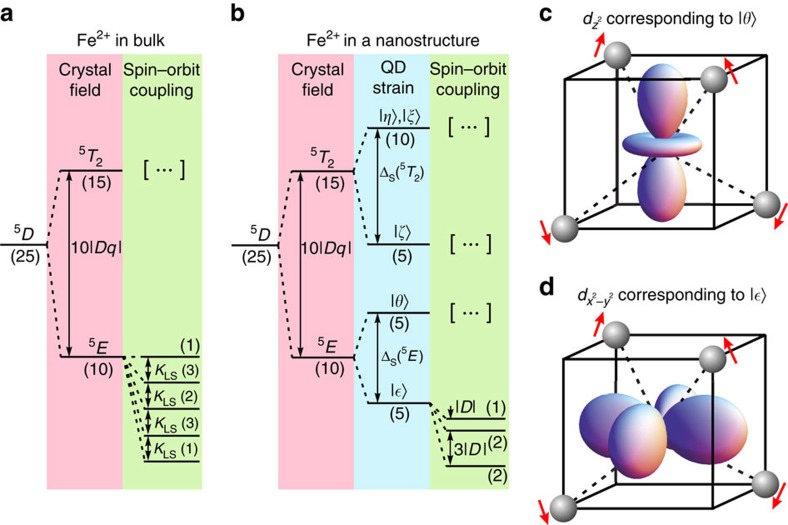
Energy levels of the Fe^2+^ ion in crystal environment. Energy spectra of the Fe^2+^ ion in (**a**) a bulk zinc-blende semiconductor and (**b**) a nanostructure with a strong in-plane compressive strain. Numbers in parentheses denote the degeneracy of the energy levels. Six-electron Fe^2+^ orbital states split by the QD strain are denoted as 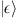, |*θ*〉, |*ζ*〉, |*η*〉 and |*ξ*〉 after Vallin *et al*.^16^ (these orbital states can be also referred to as single-electron *d* orbitals of corresponding symmetries, as described in Supplementary Table 1). (**c**,**d**) Visualization of |*θ*〉 and 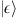 orbital states forming the ^5^*E* subspace with single-electron 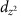 and 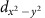 orbitals of the same symmetries. Arrows schematically mark the shift of the neighbouring anions owing to the strain of the QD.

The dominant effect defining energy spectrum of a transition metal ion in the bulk semiconductor is the crystal field^2^,^20^. Fe^2+^ has configuration *d*^6^, which means that the *d*-shell electrons have combined orbital angular momentum of *L*=2 and spin of *S*=2. The crystal field affects only orbital part of the wave function and splits five orbital states of the ion into two subspaces: twofold degenerate ^5^*E* and threefold degenerate ^5^*T*_2_, with ^5^*E* being lower energy in *T*_d_ symmetry (Fig. 1a). For Fe^2+^ in CdSe or ZnSe this splitting is about 10|*Dq*|≈0.3 eV (refs 6, 22, 44). Thus, the ^5^*T*_2_ level is not populated even at room temperature and the properties of the Fe^2+^ ion are defined only by the states in the ^5^*E* subspace. These states are not affected by a static Jahn–Teller distortion, as it was shown for many Fe-doped semiconductors^5^,^14^,^15^,^16^. Consequently, the second effect in order of strength is the spin–orbit interaction *λ***LS**. It results in splitting of ^5^*E* levels into five equidistant groups, as shown in Fig. 1a. The value of the splitting *K*_LS_ is determined by the effective strength *λ* of the spin–orbit interaction and the crystal field splitting 10|*Dq*| according to *K*_LS_=6*λ*^2^/10|*Dq*|≈2 meV (refs 19, 21). The presence of a dynamical Jahn–Teller effect or application of additional stress in experimentally accessible range results in only small shifts of those energy levels and can be treated perturbatively^21^,^22^,^45^,^46^,^47^,^48^. In any case, the lowest energy group consists of a single nondegenerate state, which determines the nonmagnetic character of the Fe^2+^ ion ground state.

We find that strong structural strain of a QD changes hierarchy of the Fe^2+^ energy scales known from the bulk. The dominant effect is still the crystal field, but the second effect becomes the biaxial strain. It lifts orbital degeneracy of the ^5^*E* subspace, splitting it into 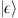 and |*θ*〉 states of symmetries corresponding to single-electron 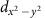 and 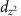 orbitals, respectively (Fig. 1b). The ordering of those states is determined by the sign of the strain, which, given the CdSe/ZnSe lattice mismatch, has compressive character. Qualitatively, such strain pulls the tetrahedral lattice bonds away from *x–y* plane (for details, see Supplementary Fig. 1) and thus lowers the energy of the 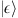 orbital while increasing the energy of the |*θ*〉 one (as schematically depicted in Figs 1c,d). More strict analysis leading to the same-level ordering is presented in Supplementary Note 1. This analysis reveals also that the strain-induced splitting of the Fe^2+^ states significantly exceeds the strength of the spin–orbit interaction, which determines the spin part of the wave function. The spin–orbit interaction contributes to the energy of spin states of 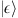 orbital in the second order. It favours high-spin states according to the effective spin Hamiltonian 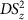 with *D*<0 (for details, see Supplementary Note 2). In this approximation, (within the discussed second order of the *λ***LS** interaction) the ground state is doubly degenerate with the spin part of *S*_*z*_=±2. Since the integer spin of the Fe^2+^ ion does not warrant exact Kramers degeneracy, further analysis and experimental results presented below reveal that two low-energy states are in fact separated by small energy, but still they can be easily split by an external magnetic field, in a clear contrast to previously described case of the Fe^2+^ embedded in bulk semiconductor.

### Photoluminescence of a strained QD with a single Fe^2+^ ion

The experiment evidencing actual behaviour of the Fe^2+^ ion in a strained nanostructure is carried out on a number (>30) of single QDs, each incorporating an individual iron ion. Such structures are presented here for the first time. Self-assembled zinc-blende CdSe:Fe QDs in ZnSe barrier are grown using molecular beam epitaxy. Low-temperature (down to 1.5 K) photoluminescence experiments on individual QDs are performed in a setup providing spatial resolution of 0.5 μm without the need for mesas or masks. More details on sample preparation and experiment are given in the Methods section.

**Figure 2:**
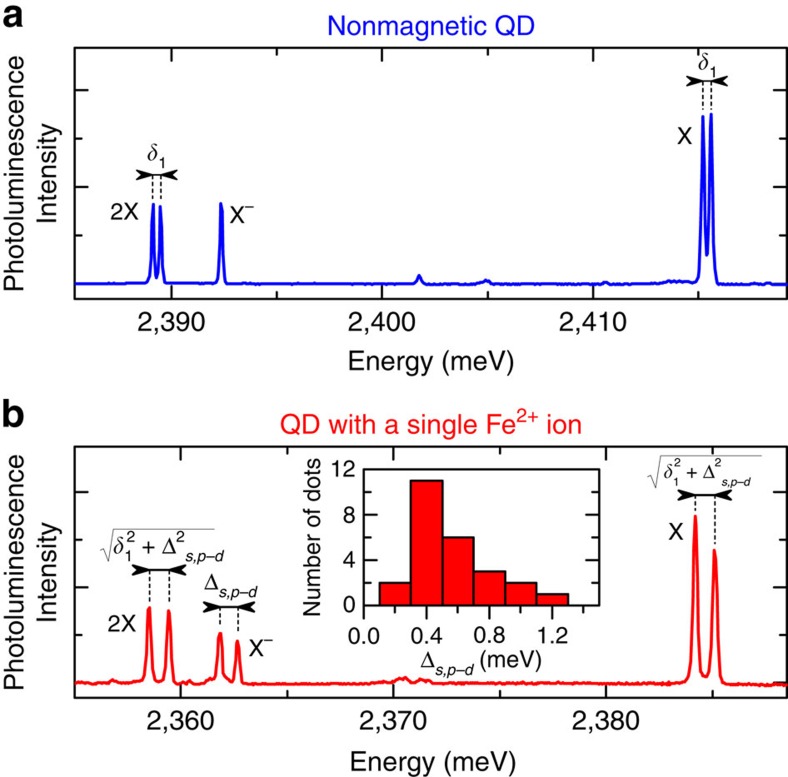
Photoluminescence spectra of CdSe/ZnSe QDs with and without a single Fe^2+^ ion. (**a**) A photoluminescence spectrum of a typical CdSe QD showing X, X^−^ and 2X lines. Neutral complexes exhibit anisotropic splitting of *δ*_1_=370 μeV. (**b**) A photoluminescence spectrum of a QD with a single Fe^2+^ ion. The photoluminescence lines are split mainly owing to *s*,*p*−*d* exchange interaction between confined carriers and the *d*-shell electrons of the ion. For both spectra continuous background was subtracted. Inset is a histogram of the *s*,*p*−*d* exchange splitting of the X^−^ emission line. The cutoff at Δ_*s*,*p*−*d*_≲0.3 meV is because of our selection procedure, which is limited by the resolution of our experimental setup—only dots with larger zero-field splitting were tested in the magnetic field to verify the presence of the Fe^2+^ ion.

As expected for random character of low-density doping, in the same sample we find QDs incorporating single Fe^2+^ ions and undoped QDs for reference purposes. Photoluminescence spectra corresponding to both of these cases are shown in Fig. 2. Figure 2a presents a typical spectrum of an undoped QD. The spectrum exhibits all standard features of epitaxial QDs^32^,^49^,^50^,^51^. The sharp emission lines originate from recombination of different excitonic complexes, including neutral exciton (X), negatively charged exciton (X^−^) and biexciton (2X). The X and 2X lines are split owing to anisotropic part of electron-hole exchange interaction. In the case of QD shown in Fig. 2a this splitting yields *δ*_1_=370 μeV, which is a typical value for self-assembled CdSe/ZnSe QDs^50^,^51^. On the other hand, the charged exciton line does not exhibit any splitting, in accordance with the Kramers rule for systems with odd number of fermions.

In comparison, introduction of a single Fe^2+^ ion into a QD leads to distinctive changes in the photoluminescence spectrum, as shown in Fig. 2b. The emission lines still correspond to recombination of the same excitonic complexes, however, their structure is determined by the *s*,*p*−*d* exchange interaction with the resident ion. The main effect is a strong splitting of each of the observed emission lines. It is particularly striking for the typically degenerate charged exciton, but also for the neutral exciton it is significantly stronger than the typical value of *δ*_1_. Such a physical picture is similar for a large number of studied Fe-doped QDs, as proven by distribution of measured *s*,*p*−*d* exchange splittings presented in the inset of Fig. 2b. The presence of such *s*,*p*−*d* splitting is a direct confirmation of the magnetic character of the Fe^2+^ ion. It originates from the fact that the Fe^2+^ spin may be aligned either parallel or anti-parallel to the exciton angular momentum, which would not be possible in the case of nonmagnetic ground state.

### Magneto-photoluminescence of a QD with a single Fe^2+^ ion

**Figure 3:**
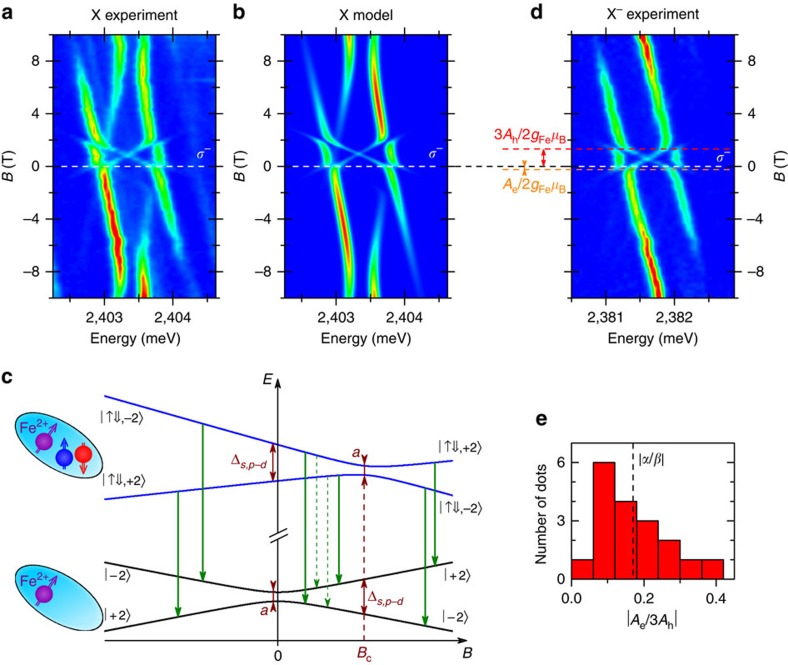
Magneto-optical spectroscopy of X and X^−^ in a QD with a single Fe^2+^ ion. Magnetic field dependence of the photoluminescence spectrum of a X: (**a**) experimental data and (**b**) simulation assuming strain-induced magnetism of the Fe^2+^ ion, as described in the text. The spectra were measured and simulated in *σ*^−^ circular polarization. (**c**) Schematic field dependence of the initial and final energy levels of the X recombination together with the indicated *σ*^−^-polarized X optical transitions observed in photoluminescence measurements. The upper pair of levels corresponds to 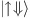 exciton coupled with the ion spin (where ↑ and 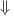 represent the spin projection of the electron and the heavy hole on the growth axis, respectively), while the bottom pair represents the energies of the ion states in the empty dot. The excitonic transitions preserving (altering) the ion spin projection are marked with solid (dashed) arrows. (**d**) Magnetic field evolution of a X^−^ photoluminescence spectrum measured in *σ*^−^ circular polarization. Red and orange dashed lines indicate magnetic field values *B*=3*A*_h_/(2*g*_Fe_*μ*_B_) and *B*=*A*_e_/(2*g*_Fe_*μ*_B_), respectively, which correspond to the end points of the cross-like feature in X^−^ magneto-photoluminescence. (**e**) Histogram of experimentally determined ratio of ion-electron to ion-hole exchange integrals for the Fe^2+^ in a QD. Dashed line indicates the ratio |*α*/*β*| of *s-d* and *p-d* exchange constants known from the bulk Cd_1−*x*_Fe_*x*_Se.

In order to provide the final proof of the magnetic character of the Fe^2+^ ion in a QD, we measured the evolution of the X photoluminescence spectrum in external magnetic field applied along the growth direction (quantization axis of the magnetic ion and QD excitons). Typical results obtained in *σ*^−^ polarization of detection are shown in Fig. 3a. We note that the observed pattern is quite similar to the one obtained for InAs/GaAs QD containing a complex of a single Mn^2+^ ion and a bound hole^27^,^31^,^39^,^52^,^53^, despite different electronic and spin configurations.

Magneto-photoluminescence results in Fig. 3a seem complex, however, they originate from quite simple behaviour of the initial and final energy levels of the transitions, as illustrated in Fig. 3c. First effect of the magnetic field is the Zeeman splitting between *S*_*z*_=2 and *S*_*z*_=−2 states of the Fe^2+^ ion. Unfortunately, the photoluminescence spectrum does not show this splitting directly, since, in general, exciton recombination does not affect the ion spin state and thus the energy of emitted photon does not depend on the ion Zeeman splitting. However, in the vicinity of the level anticrossings the Fe^2+^ spin states are mixed and this selection rule is relaxed. Indeed, data in Fig. 3a feature several weaker lines in the anticrossing range (that is, 0–2 T). Before we discuss the origin of the anticrossings, let us analyse the behaviour of these weak lines, in particular the cross-like feature. The two crossing lines correspond to transitions involving the change of the ion spin from *S*_*z*_=±2 to *S*_*z*_=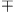
2. The splitting between them depends almost linearly on the magnetic field with a slope of about 0.84 meV T^−1^. More precise fitting including non-linearity owing to proximity of the anticrossings gives slightly larger value of 0.92 meV T^−1^. Taking into account that |Δ*S*_*z*_|=4 for both the initial and the final states, this slope corresponds to g-factor *g*_Fe_=2.0, exactly as expected for the Fe^2+^ spin.

Let us now focus on the nature of the observed anticrossings. The first, relatively weak anticrossing occurs around 0 T. It is a signature of the fact that the *S*_*z*_=±2 states of the Fe^2+^ ion are not perfectly degenerate, but are split by a small energy *a*, as shown in Fig. 3c. This splitting varies between different studied dots and its typical value yields about 50 μeV. Consequently, this splitting is much smaller than the X–Fe^2+^ exchange or Zeeman energy above 1 T. Such a splitting arises because of the spin–orbit coupling, acting either in the fourth order, or lower orders in the presence of an in-plane anisotropy of the QD^27^,^53^,^54^ (for details, see Supplementary Note 2). In both cases, the resulting zero-field eigenstates of the Fe^2+^ are 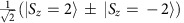.

The second anticrossing around 2 T is closely related to the first one. It occurs when the effective magnetic field acting on the Fe^2+^ spin in the presence of the *σ*^−^-emitting exciton vanishes. Since exchange field of this exciton increases the energy of the state corresponding to *S*_*z*_=−2 ion spin projection (Fig. 3c), the anticrossing of the Fe^2+^ ion is effectively shifted from 0 T to a higher field.

Finally, there is also the third, stronger anticrossing around ±9 T. This anticrossing is observed for both negative and positive magnetic field (or equivalently: in *σ*^+^ and *σ*^−^ polarization), which clearly indicates that it is owing to mixing of the exciton part of the total wave function. Indeed, the states involved in the anticrossing correspond to *σ*^−^-and *σ*^+^-emitting excitons coupled with *S*_*z*_=−2 spin projection of the Fe^2+^ ion (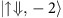 and 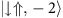). The anticrossing occurs when the excitonic Zeeman effect reduces the ion-related exchange splitting of the involved states and the anisotropic electron-hole exchange interaction becomes dominant source of the splitting. It should be noted that this anticrossing does not mix different states of the Fe^2+^ ion and thus in this range of magnetic field the optical recombination preserves the spin of the ion.

In order to quantitatively verify our interpretation of the magneto-photoluminescence results, we performed a numerical simulation of the expected field dependence of X photoluminescence spectrum. The simulation is based on the spin Hamiltonian of an ion-exciton system described in the Methods section. As shown in Fig. 3b, such a model reproduces all features of the experimental results. The simulation correctly captures even the observed thermalization of the ion spin at increasing magnetic field by taking into account the effective Fe^2+^ spin temperature of 15 K. Such a good overall agreement provides a strong proof of correct identification of all relevant effects.

In the experiments reported so far we have used the X to probe the properties of the Fe^2+^ ion. To determine both exchange integrals between the ion and each confined carrier (the electron and the hole), one needs to probe the Fe^2+^ ion using single carriers. Experimentally, it is realized with a negatively charged exciton (refs 27, 53), the magneto-photoluminescence of which is presented in Fig. 3d. In the initial state of X^−^ recombination the Fe^2+^ interacts only with the hole (owing to spin-pairing of the two electrons), while in the final state the ion interacts only with the remaining electron. As such, the Fe^2+^ ion experiences an exchange field in both of these states, which reduces a mixing between the Fe^2+^ spin states *S*_*z*_=±2 at *B*=0 T. To bring these two spin states into an anticrossing, one needs to apply a magnetic field, which compensates either ion-hole or ion-electron exchange interaction (in the initial or final state of the X^−^ optical transitions). Such values of the magnetic field correspond to the end points of the cross-like feature in X^−^ magneto-photoluminescence, which is thus shifted with respect to previously considered case of the X, as seen in Figs 3a,d. Indeed, the cross-pattern for the X begins at zero magnetic field, while the end points of this pattern for the X^−^ are given by *B*=*A*_e_/(2*g*_Fe_*μ*_B_) and *B*=3*A*_h_/(2*g*_Fe_*μ*_B_), where *A*_e_ and 3*A*_h_ denote the ion-electron and ion-hole exchange integrals, respectively. Both of these integrals might be thus directly determined based on the magnetic field dependence of the X^−^ photoluminescence spectrum. The results of such an analysis performed for a number of QDs are presented in Fig. 3e. They evidence that ion-hole exchange constant clearly dominates over one of ion-electron interaction. It can be directly related to the differences in values of bulk Cd_1−*x*_Fe_*x*_Se *s-d* and *p-d* exchange constants^55^ *N*_0_*α*=0.26 eV and *N*_0_*β*=−1.53 eV. In particular, the ratio *A*_e_/3*A*_h_ should be equal to ratio *α*/*β* if only the local densities of electron and hole wave functions at the Fe^2+^ site in the QD are equal. Centring of the results in the Fig. 3e around a marked *α*/*β* ratio is fully consistent with this prediction.

It is noteworthy that the field evolution of X^−^ photoluminescence spectrum does not feature an anticrossing at high-magnetic fields, which arises for the X because of the electron-hole exchange interaction. This observation is a direct confirmation that such an interaction affects neither the initial nor the final state of the X^−^ recombination.

### Optical orientation of the spin of a single Fe^2+^ ion

Zero-field splitting of the Fe^2+^ spin may raise concerns whether the iron spin can be controlled optically. In order to address this issue we performed a proof-of-concept measurement of the optical orientation^35^,^36^,^37^,^38^,^39^,^40^. Experimentally, we induce a polarization of the Fe^2+^ spin by injecting spin-polarized excitons to the QD under circularly polarized non-resonant excitation (at *E*=2.54 eV). The spin state of the Fe^2+^ ion is monitored using the relative intensities of the photoluminescence lines of the 2X. The advantage of using the 2X instead of the X as a probe of the ion spin state lies in the fact that the 2X is a spin-singlet and therefore allows us to circumvent the issue of excitonic spin relaxation^40^.

**Figure 4:**
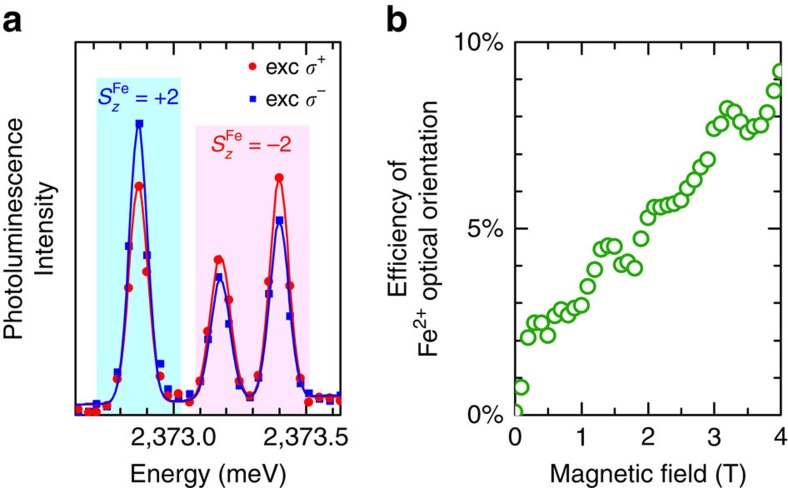
Optical orientation of the Fe^2+^ spin. (**a**) Photoluminescence spectra of the 2X measured in *σ*^−^ polarization at *B*=4 T using circularly polarized excitation of different helicity (solid lines represent multi-peak Gaussian fits to the measured spectra). Upon *σ*^+^-polarized excitation the emission line corresponding to the Fe^2+^ spin projection *S*_*z*_=2 is weaker, while the two lines corresponding to *S*_*z*_=−2 are stronger as compared with the *σ*^−^-polarized excitation. The lines corresponding to *S*_*z*_=−2 are mixed owing to anisotropic fine structure of the exciton, which does not affect the Fe^2+^ spin. (**b**) Efficiency of optical orientation of the Fe^2+^ spin as a function of the magnetic field (the data points were averaged over several measurements of the 2X photoluminescence spectra at each field).

The intensities of the 2X lines corresponding to different Fe^2+^ spin states vary depending on the polarization of the excitation, as shown in Fig. 4a presenting the 2X photoluminescence spectra measured at *B*=4 T. Such a variation can be explained only by the optically induced change in the occupation of the Fe^2+^ states and thus is a direct evidence of an optical control over the Fe^2+^ spin. The comparison of the two spectra allows us to quantify the effect of the polarization of the excitation. In the presented case, switching the polarization from *σ*^+^ to *σ*^−^ changes the mean spin of the Fe^2+^ by Δ〈*S*_*z*_〉≈0.4, that is by about 10%. Similar efficiency was observed previously for non-resonant pumping of the Mn^2+^ spin in CdSe QDs^40^ and its value is most likely limited by depolarization of the itinerant carriers during the energy relaxation towards the ground state in a QD.

Figure 4b shows the dependence of the optical spin orientation efficiency on the magnetic field. It exhibits a pronounced close-to-linear increase, which is similar as in the case of previously studied Mn^2+^ ion^40^. However, for the Fe^2+^ we also observe a sharp drop of the orientation efficiency at *B*=0 T. This effect is a result of a mismatch between *σ*^±^-polarized pumping and the mixed character of Fe^2+^ spin states in the absence of the magnetic field. In other words, the ion spin-polarization induced by the exciton cannot be maintained after the recombination, since the Fe^2+^ spin undergoes oscillations between two mixed eigenstates at *B*=0 T. Consequently, future experiments on zero-field optical manipulation of the Fe^2+^ spin should use a resonant initialization protocol and temporally resolved spin readout^37^,^42^ rather than rely on continuous-wave non-resonant pumping with circularly polarized light.

## Discussion

All the presented results clearly show that the structural strain of the QD induces magnetic character of the Fe^2+^ ion in its ground state. Thus, a CdSe QD with a single Fe^2+^ ion joins other solotronic QD systems like CdTe, InAs and CdSe QDs doped with single manganese or cobalt ions^26^,^27^,^32^, which grant optical access to the spin degree of freedom of a single transition metal ion. In contrast to rich, but vulnerable *S*=5/2 Mn^2+^ spin in CdTe and CdSe QDs, the Fe^2+^ in a CdSe dot can be regarded as a robust two-level system. Its spectroscopic properties are very similar to InAs QD doped with a single Mn^2+^ ion antiferromagnetically coupled to a hole^27^,^31^,^39^,^52^,^53^. In both cases, the magnetic field dependence of the excitonic photoluminescence spectrum is a result of an interplay between the uniaxial anisotropy of the ion spin, its zero-field splitting and the Ising coupling with the excitons. This observation is valid regardless of the differences between these systems, such as different electronic configuration of the ions or differences in possible mechanisms leading to the zero-field splitting of the ion states. The latter differences stem mostly from different effective spins of the two ions: *S*=2 in case of the Fe^2+^ and *J*=1 for the Mn^2+^-hole complex. As such, the Fe^2+^ ground states are associated with a larger difference Δ*S*_*z*_=4, and thus they cannot be coupled by an in-plane anisotropy 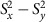 acting in the first order. Practical conclusion from this consideration is that the Fe^2+^ spin is promising as a two-level system since it offers additional protection from the perturbations in the environment. On top of that, CdSe/ZnSe QDs with single Fe^2+^ ions, in contrast to the systems mentioned earlier, can be grown free of any nuclear spins: the natural iron has nuclear spin *I*=0 and all the elements of the host lattice have significant abundance of isotopes without nuclear spins. Altogether these properties make the Fe^2+^ in CdSe QDs an ideal system for quantum information technology. However, the importance of our results is not limited to this particular system. It is a general example of the fact that even well-established textbook knowledge of energy spectrum of various dopants should be carefully re-evaluated in the world of semiconductor nanostructures.

## Methods

### Sample preparation

Samples with self-assembled zinc-blende CdSe:Fe QDs in ZnSe barrier are grown using molecular beam epitaxy double-chamber system manufactured by SVT Associates. About two monolayers of CdSe:Fe are deposited without any growth interruptions on 1.5-μm thick ZnSe buffer grown on GaAs (100) substrate. QDs are covered by a 50-nm thick ZnSe cap layer. Iron doping density is adjusted in order to optimize the probability of finding a QD with exactly one Fe^2+^ ion.

### Experiment

Photoluminescence experiments on individual QDs are performed in a micro-photoluminescence setup providing a spatial resolution of 0.5 μm. Low-temperature (down to 1.5 K) photoluminescence is excited non-resonantly either at 405 or 488 nm in the case of the measurements of optical spin orientation of the Fe^2+^ ion. The sample is placed in the split-coil superconducting magnet producing a magnetic field of up to 10 T either in Faraday or Voigt configuration.

### Modelling of magneto-photoluminescence

The X magneto-photoluminescence from Fig. 3a is quantitatively described by a model of a X inside a QD with a single Fe^2+^ ion. Our simulation is based on the standard procedure of finding the eigenstates of the exciton complex and the empty (that is, without the exciton) QD and subsequent calculation of allowed optical transitions. For simplicity, we assume that the spatial part of excitonic wave function is not substantially modified by the magnetic field and restrict our analysis to the spin degree of freedom. The possible initial states of X–Fe^2+^ complex are found by diagonalization of the Hamiltonian^26^,^27^,^32^:


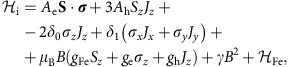


where **S** and ***σ*** are the spin operators of the Fe^2+^ and the electron, while ***J*** is effective 1/2 spin operator in the two-dimensional subspace of lowest-energy heavy hole states. The first two terms in 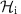 describe the *s*, *p*−*d* exchange interaction between the iron ion and confined carriers^55^,^56^,^57^,^58^,^59^ with *A*_e_ and 3*A*_h_ being the ion-electron and ion-hole exchange integrals. The next two terms represent isotropic and anisotropic contributions to the electron-hole exchange interaction^49^ with characteristic energies of *δ*_0_ and *δ*_1_. The parameters *g*_Fe_, *g*_e_ and *g*_h_ are g-factors of the Fe^2+^ ion, electron and hole, respectively, while *γ* is the excitonic diamagnetic shift constant. The last term 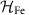 is the Hamiltonian of the Fe^2+^ ion leading to energy spectrum as in Fig. 1b. Within the lowest-energy orbital state subspace, the spin part of 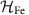 might be expressed as


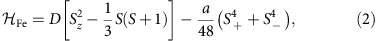


where the first term leads to a splitting of three spin subspaces corresponding to different |*S*_*z*_|, while *a* denotes a small splitting between the two Fe^2+^ lowest energy spin states 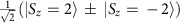. This splitting is directly determined from the photoluminescence studies and yields typically about 50 μeV. Conversely, the value of |*D*| is much larger, as we find no spectral signatures of |*S*_*z*_|<2 states in our experiments. A quantitative estimation of the value of this splitting 3*D*<−2.5 meV is obtained in Supplementary Note 3 based on the measurements of X magneto-photoluminescence in Voigt configuration (the results of which are shown in Supplementary Fig. 2).

The oscillator strengths of X optical transitions are calculated between eigenstates of the Hamiltonian 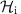 and the Hamiltonian 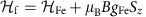 describing the final state of the recombination (that is, an empty QD). The relevant parameters of both of these Hamiltonians are found by fitting the field dependence of the photoluminescence spectrum. Additional parameter *E*_0_ is introduced later to plot the simulation in the same energy range as the experimental results. The optical spectrum is simulated as a series of peaks at energies corresponding to possible transitions between possible initial and final states. The intensity of a given peak is the product of calculated oscillator strength and the Boltzmann factor corresponding to thermalization of the ion spin at an effective temperature of 15 K. For the sake of the presentation, the peaks are plotted as gaussians with FWHM of 0.1 meV. Strictly speaking, such procedure gives us the low-power absorption spectrum, but it is also similar to the photoluminescence spectrum if only the exciton states have the same lifetime and if it is shorter than the thermalization of the excited state.

## Additional information

**How to cite this article:** Smoleński, T. *et al*. Magnetic ground state of an individual Fe^2+^ ion in strained semiconductor nanostructure. *Nat. Commun.* 7:10484 doi: 10.1038/ncomms10484 (2016).

## Supplementary Material

Supplementary Information Supplementary Figures 1-2, Supplementary Table 1, Supplementary Notes 1-3 and Supplementary References
