# Supplementary material for: Magnetic ground state of an individual Fe2+ ion in strained semiconductor nanostructure
Source: Nat Commun. 2016 Jan 28;7:10484. doi: 10.1038/ncomms10484 (PMC4738340; doi:10.1038/ncomms10484)
Supplement: Supplementary Information — Supplementary Figures 1-2, Supplementary Table 1, Supplementary Notes 1-3 and Supplementary References [file ncomms10484-s1.pdf]

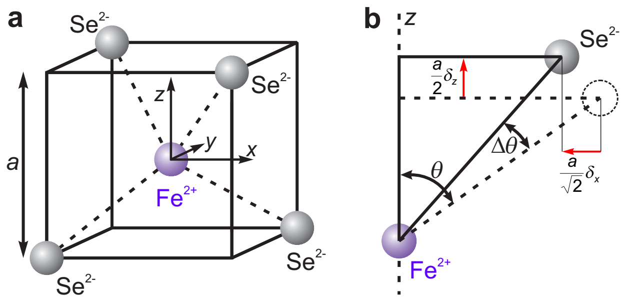

Supplementary Figure 1. Relative position of ions in the crystal lattice. (**a**) A schematic visualisation of Fe^{2+} ion surrounded tetrahedrally by four Se^{2-} anions occupying alternating corners of a cubic elementary cell. (**b**) Illustration of strain-induced displacement of each Se^{2-} anion (not in scale). The angle θ corresponds to the anion position in pure T_{d} symmetry, while $\Delta\theta$ denotes the change arising due to the strain.

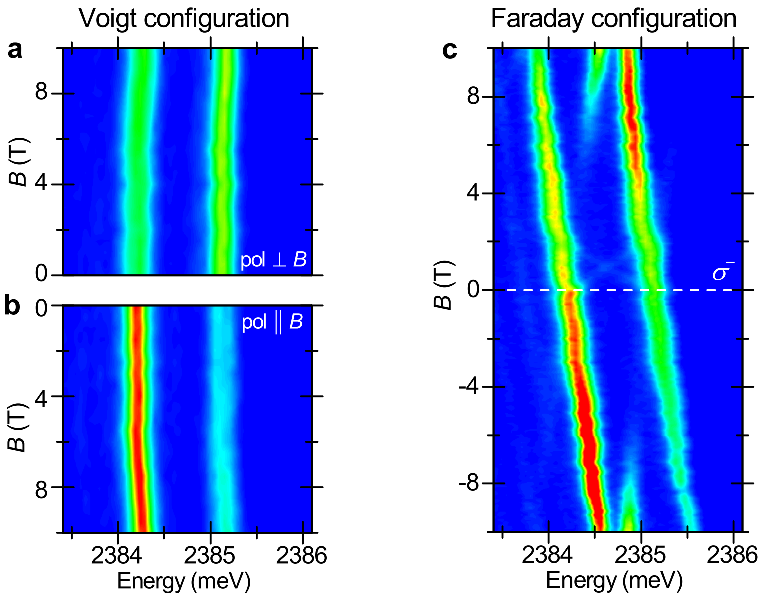

Supplementary Figure 2. Magneto-photoluminescence of a CdSe/ZnSe QD with a single Fe^{2+} ion. Magnetic field dependence of the neutral exciton PL spectrum measured in (**a**,**b**) Voigt and (**c**) Faraday configurations for the same QD with a single Fe^{2+} ion. The spectra in (**a**,**b**) were detected in linear polarizations of orientation perpendicular or parallel to the field direction, while the spectra in (**c**) were measured in σ^- circular polarization.

Subspace	Orbital wave function	Corresponding single-electron orbital
$^5T_2$	$\|\eta\rangle = -(Y_2^1 - Y_2^{-1})/\sqrt{2}$	d_{xz}
	$\|\xi\rangle = i(Y_2^1 + Y_2^{-1})/\sqrt{2}$	d_{yz}
	$\|\zeta\rangle = i(Y_2^2 - Y_2^{-2})/\sqrt{2}$	d_{xy}
^5E	$\|\theta\rangle = Y_2^0$	d_{z^2}
	$\|\epsilon\rangle = (Y_2^2 + Y_2^{-2})/\sqrt{2}$	$d_{x^2-y^2}$

Supplementary Table 1. Symmetry of five multi-electron orbital states of the Fe^{2+} ion. Since six d-shell electrons have total orbital angular momentum $L = 2$, their orbital wave functions transform like linear combinations of spherical harmonics $Y_2^{L_z}$, where $L_z = 0,\ \pm1,\ \pm2$. They are explicitly given in the second column and denoted by symbols η, ξ, ζ, θ, ϵ after J. T. Vallin *et al.*[1]. Since d^6 configuration corresponds to half-filled shell with one additional electron, these multi-electron orbital states may be also discussed using single-electron d orbitals of the same symmetries, which are listed in the last column.

Supplementary Note 1. Energy levels of Fe^{2+} ion in the presence of biaxial strain

In order to estimate the magnitude of strain-induced splitting of ${}^5E$ orbitals we employ a simple point-charge model, in which the crystal field acting on the single Fe^{2+} ion comes from four point charges corresponding to neighboring Se^{2-} anions (Supplementary Fig. 1a). The electrostatic potential from these charges can be expanded in multipole series. In the case of pure tetrahedral $T_{\rm d}$ symmetry, the terms relevant to d orbitals of the Fe^{2+} ion are given by[2–4]

$$V_{\rm C}(\vec{r}) = A\frac{r^4}{d^4}\left[Y_4^0 + \sqrt{\frac{5}{14}}\left(Y_4^4 + Y_4^{-4}\right)\right], \tag{1}$$

where $Y_n^m(\theta,\varphi)$ are spherical harmonics in a coordinate system (r,θ,φ) centered on the Fe^{2+} ion. The coefficient A denotes the strength of the crystal field and is given by $A = -\frac{56\sqrt{\pi}}{27}\frac{e^2}{4\pi\epsilon_0\epsilon_{\rm r}d}$, where $\epsilon_{\rm r}$ is the relative permittivity of CdSe taken as 9.2 (ref. 5) and $d = a\sqrt{3}/2$ is the distance between Fe^{2+} and each anion in the crystal lattice (with $a \approx 6.08$ Å being zinc-blende CdSe lattice constant[6]).

The structural strain of a CdSe/ZnSe QD shifts the anions away from their normal positions and thus lifts the tetrahedral symmetry. The main factor determining the character of the strain is a large lattice mismatch between CdSe and ZnSe, which is equal to about 7% (refs. 6,7). As a result, the dot material is compressed in xy plane and stretched along the growth direction z. We assume that the relative reduction of a lattice constant is the same for x and y directions and equal to δ_x. Consequently, the relative elongation of an elementary cell in z is given by $\delta_z = 2\nu\delta_x$, where $\nu \approx 0.35$ is CdSe Poisson's ratio[8]. Under these assumptions, the angular coordinate φ of each anion in xy plane remains unchanged, while the angle θ between the z axis and direction of Fe^{2+}–Se^{2-} bond is slightly decreased, as schematically shown in Supplementary Fig. 1b. To the accuracy of linear terms in δ_x, the change of θ angle is given by $\Delta\theta = \sqrt{2}(1+2\nu)\delta_x/3$, which for maximal δ_x limited by the lattice mismatch corresponds to about 3°.

Such strain-induced displacement of the anions lowers the symmetry to $D_{\rm 2d}$ and introduces new terms to the crystal field potential, which for d orbitals read

$$V_{\rm S}(\vec{r}) = A_\theta\frac{r^2}{d^2}\cdot\left[Y_2^0 - \frac{5\sqrt{5}}{27}\frac{r^2}{d^2}\left(Y_4^0 - \sqrt{\frac{7}{10}}\left(Y_4^4 + Y_4^{-4}\right)\right)\right], \tag{2}$$

where $A_\theta \approx 16\sqrt{\frac{2\pi}{5}}\frac{e^2}{4\pi\epsilon_0\epsilon_{\rm r}d}\Delta\theta$. The overall potential $V_{\rm C} + V_{\rm S}$ splits fivefold degenerate orbitals of the Fe^{2+} ion into four subspaces, which energies take a form

$$E(|\eta\rangle) = E(|\xi\rangle) = -4Dq + Ds + 2Dt, \tag{3}$$
$$E(|\zeta\rangle) = -4Dq - 2Ds - 4Dt, \tag{4}$$
$$E(|\theta\rangle) = 6Dq + 2Ds - 3Dt, \tag{5}$$
$$E(|\epsilon\rangle) = 6Dq - 2Ds + 3Dt, \tag{6}$$

where $10|Dq|$ (with $Dq < 0$) is a splitting between ${}^5T_2$ and ${}^5E$ subspaces arising only due to the crystal field of pure $T_{\rm d}$ symmetry, while Ds and Dt are the splittings induced by the strain. They are given by

$$Dq = -\frac{4}{27}\frac{e^2}{4\pi\epsilon_0\epsilon_{\rm r}d^5}\langle r^4\rangle, \tag{7}$$
$$Ds = \frac{16}{21}(1+2\nu)\delta_x\frac{e^2}{4\pi\epsilon_0\epsilon_{\rm r}d^3}\langle r^2\rangle, \tag{8}$$
$$Dt = \frac{160}{567}(1+2\nu)\delta_x\frac{e^2}{4\pi\epsilon_0\epsilon_{\rm r}d^5}\langle r^4\rangle, \tag{9}$$

where $\langle r^n\rangle$ denotes the expectation value of nth power of Fe^{2+} orbital radius r. According to Eqs. (5) and (6), the strain-induced energy splitting of orbitals $|\epsilon\rangle$ and $|\theta\rangle$ forming the ${}^5E$ subspace corresponds to $\Delta_{\rm S}({}^5E) = 4Ds - 6Dt$. Under an assumption that $\langle r^n\rangle \approx \langle r\rangle^n$ it can be expressed using $|Dq|$ and reads

$$\Delta_{\rm S}({}^5E) = (1+2\nu)\delta_x\frac{80}{7}|Dq|\cdot\left(\frac{2\sqrt{3}}{5}\sqrt{\frac{e^2}{4\pi\epsilon_0\epsilon_{\rm r}d|Dq|}} - 1\right). \tag{10}$$

Since $10|Dq| \approx 0.3$ eV for CdSe[9–11], the splitting $\Delta_{\rm S}({}^5E)$ corresponds to $\delta_x\cdot 0.7$ eV, which for maximal δ_x of 0.07 yields about 50 meV. This value is significantly larger than the splitting of ${}^5E$ orbitals arising from spin-orbit coupling with

higher energy states, which was found to be of the order of a few meV in bulk semiconductors[11–13]. Consequently, our calculations reveal that the crystal field and strain are dominant effects influencing the energies of the Fe^{2+} ion states in a CdSe/ZnSe QD and the spin-orbit interaction might be treated perturbatively. Moreover, our results also confirm that $|\epsilon\rangle$ state is the lowest-energy orbital state of this ion embedded in a dot, since the computed $\Delta_{\mathrm{S}}(^5E)$ is positive for the compressive strain (that is for $\delta_x > 0$). It should be noted that the actual value of this splitting may be deviated to a some degree from the one given by Eq. (10), since the exploited point-charge model neglects the covalency of bonds between magnetic ion and anions in the crystal lattice. Nevertheless, as proven by the experiment, the general conclusions of our analysis are not altered by this simplification, even though it is probably quite important, especially in the case of II-VI compounds studied in this work.

Supplementary Note 2. Effects of spin-orbit coupling

Since both the crystal field and strain does not affect the spin part of the Fe^{2+} ion states, the lowest-energy orbital $|\epsilon\rangle$ is fivefold degenerate due to the ion spin $S = 2$. This degeneracy is partially lifted by the spin-orbit interaction $\lambda\mathbf{LS}$. Such interaction splits the spin states of $|\epsilon\rangle$ orbital in the second order through mixing with higher energy orbitals forming the ${}^5T_2$ subspace. This splitting can be expressed by the effective spin Hamiltonian:

$$\mathcal{H}_{\mathrm{LS}} = D\left[S_z^2 - \frac{1}{3}S(S+1)\right], \tag{11}$$

where D is given by

$$D = \frac{\lambda^2}{E(|\eta\rangle) - E(|\epsilon\rangle)} - \frac{4\lambda^2}{E(|\zeta\rangle) - E(|\epsilon\rangle)}. \tag{12}$$

Taking into account Eqs. (3)-(6) and bearing in mind that Ds and Dt are positive for the compressive strain, one obtains $E(|\eta\rangle) > E(|\zeta\rangle) > E(|\epsilon\rangle)$, which yields $D < -3\lambda^2/[E(|\zeta\rangle) - E(|\epsilon\rangle)] < 0$. Consequently, the states corresponding to $S_z = \pm 2$ spin projections have lowest energy. Their degeneracy is however lifted, when the spin-orbit coupling is considered in the higher order. In particular, in the fourth order the states $|S_z = \pm 2\rangle$ are split by $\lambda\mathbf{LS}$ interaction into linear combinations $\frac{1}{\sqrt{2}}(|S_z = 2\rangle \pm |S_z = -2\rangle)$, with the state corresponding to "+" being lower energy. The corresponding energy splitting a is given by

$$a = \frac{36\lambda^4}{\left[E(|\eta\rangle) - E(|\epsilon\rangle)\right]^2 \Delta_{\mathrm{S}}({}^5E)}. \tag{13}$$

The value of this splitting obtained for maximal strain (that is $\delta_x = 0.07$) and $\lambda \approx -10$ meV (refs. 9,13) is equal to about 55 μeV, which is consistent with our experimental results. Note, however, that additional contribution to this splitting might also arise due to in-plane anisotropy of the QD.

Supplementary Note 3. Estimation of D parameter for the Fe^{2+} ion

As we discuss in the manuscript, the ground energy level of the Fe^{2+} ion in a QD is comprised of $S_z = \pm 2$ states. According to Eq. (11), the next excited states corresponding to $S_z = \pm 1$ are separated by the energy of $3|D|$. In the optical experiments we find no signatures of these excited states, which indicates that they are not populated at cryogenic temperatures. On this bases, assuming full thermalisation of the ion spin to the bath temperature, we obtain a lower bound for $3|D| > 0.5$ meV.

A better estimation of the value of D energy is obtained from the measurements of the X PL spectrum evolution in an in-plane magnetic field applied in Voigt configuration. As shown in Supplementary Figs. 2a,b, such a magnetic field of magnitude not exceeding 10 T has no noticeable effect on the X PL spectrum, in contrast to the field applied in Faraday geometry for the same QD (Supplementary Fig. 2c). This apparent difference is directly related to a large anisotropy of the Fe^{2+} ion in the growth direction. In particular, the in-plane magnetic field couples only the Fe^{2+} states with spin projections differing by 1, which are separated by at least $|D|$. As a consequence, the influence of the in-plane field on the Fe^{2+} ion ground states remains negligible as long as the Zeeman energy $g_{\mathrm{Fe}}\mu_{\mathrm{B}}B$ is much smaller than $|D|$. More specifically, according to the 4th order perturbation theory we expect that a splitting between $\frac{1}{\sqrt{2}}(|S_z = 2\rangle + |S_z = -2\rangle)$ and $\frac{1}{\sqrt{2}}(|S_z = 2\rangle - |S_z = -2\rangle)$ states follows a formula:

$$\Delta E(B) = a + \frac{1}{12}\frac{(g_{\mathrm{Fe}}\mu_{\mathrm{B}}B)^4}{|D|^3}. \tag{14}$$

This splitting should give rise to a splitting of each X emission line into a doublet at sufficiently large B. Given that no such splitting is observed experimentally even at $B = 10$ T, we can deduce a lower bound for energy separation to the excited states $3|D| > 2.5$ meV.

Supplementary References

[1] Vallin, J. T., Slack, G. A. & Bradley, C. C. Far-infrared absorption of ZnS:Fe^{2+} in strong magnetic fields. *Phys. Rev. B* **2**, 4406–4413 (1970).

[2] Low, W. & Weger, M. Paramagnetic resonance and optical spectra of divalent iron in cubic fields. I. theory. *Phys. Rev.* **118**, 1119–1130 (1960).

[3] Slack, G. A., Roberts, S. & Vallin, J. T. Optical absorption of Fe^{2+} in CdTe in the near and far infrared. *Phys. Rev.* **187**, 511–524 (1969).

[4] Vallin, J. T., Slack, G. A., Roberts, S. & Hughes, A. E. Infrared absorption in some II-VI compounds doped with Cr. *Phys. Rev. B* **2**, 4313–4333 (1970).

[5] Geick, R., Perry, C. H. & Mitra, S. S. Lattice vibrational properties of hexagonal CdSe. *J. Appl. Phys.* **37**, 1994 (1966).

[6] Samarth, N., Luo, H., Furdyna, J. K., Qadri, S. B., Lee, Y. R., Ramdas, A. K. & Otsuka, N. Growth of cubic (zinc blende) CdSe by molecular beam epitaxy. *Appl. Phys. Lett.* **54**, 2680 (1989).

[7] McIntyre, G. J., Moss, G. & Barnea, Z. Anharmonic temperature factors of zinc selenide determined by X-ray diffraction from an extended-face crystal. *Acta Crystallogr. Sect. A* **36**, 482 (1980).

[8] Deligoz, E., Colakoglu, K. & Ciftci, Y. Elastic, electronic, and lattice dynamical properties of CdS, CdSe, and CdTe. *Physica B* **373**, 124–130 (2006).

[9] Baranowski, J. M., Allen, J. W. & Pearson, G. L. Crystal-field spectra of $3d^n$ impurities in II-VI and III-V compound semiconductors. *Phys. Rev.* **160**, 627–632 (1967).

[10] Buhmann, D., Schulz, H.-J. & Thiede, M. Zero-phonon structures in the optical spectra of some transition-metal ions in CdSe crystals. *Phys. Rev. B* **24**, 6221–6233 (1981).

[11] Malguth, E., Hoffmann, A. & Phillips, M. R. Fe in III-V and II-VI semiconductors. *Phys. Status Solidi B* **245**, 455–480 (2008).

[12] Scalbert, D., Cernogora, J., Mauger, A., Benoit à la Guillaume, C. & Mycielski, A. Resonant raman scattering on low energy excited states of Fe^{2+} in $Cd_{1-x}Fe_xSe$. *Solid State Commun.* **69**, 453 – 456 (1989).

[13] Udo, M. K., Villeret, M., Miotkowski, I., Mayur, A. J., Ramdas, A. K. & Rodriguez, S. Electronic excitations of substitutional transition-metal ions in II-VI semiconductors: CdTe:Fe^{2+} and CdSe:Fe^{2+}. *Phys. Rev. B* **46**, 7459–7468 (1992).
